# A Small-Molecular-Weight Bacteriocin-like Inhibitory Substance (BLIS) UI-11 Produced by *Lactobacillus plantarum* HYH-11 as an Antimicrobial Agent for *Aeromonas hydrophila*

**DOI:** 10.3390/vetsci12121165

**Published:** 2025-12-07

**Authors:** Yinghui He, Donghui Tang, Jiarui Lin, Jiayue Zhang, Wanli Sha, Wenlong Dong

**Affiliations:** 1 College of Animal Science and Technology, Jilin Agricultural Science and Technology College, Jilin 132101, China; heyinghui@jlnku.edu.cn (Y.H.); tangdonghui@jlnku.edu.cn (D.T.); linjiarui@jlnku.edu.cn (J.L.); zhangjiayue@jlnku.edu.cn (J.Z.); 2 Jilin Provincial Key Laboratory of Preventive Veterinary Medicine, Jilin Agricultural Science and Technology College, Jilin 132101, China; 3 Jilin Province Technology Innovation Center of Pig Ecological Breeding and Disease Prevention and Control, Jilin Agricultural Science and Technology College, Jilin 132101, China

**Keywords:** *Lactobacillus plantarum*, bacteriocins, antimicrobial mechanism, *Aeromonas hydrophila*

## Abstract

*Aeromonas hydrophila* is a typical pathogen that causes fish diseases and can easily infect different fish species. This study investigated the antibacterial activity, physicochemical properties and antibacterial mechanism of the BLIS UI-11 produced by *Lactobacillus plantarum* HYH-11, isolated from traditional kimchi in Hebei, China. It was found that BLIS UI-11 showed excellent inhibitory effect on the growth of *A. hydrophila*, and it also had a good antibacterial effect on various pathogens such as *Vagococcus fluvialis*, *Listeria monocytogenes*, *Aeromonas dhakensis*, *Aeromonas salmonicida*, *Salmonella Typhimurium*, *Escherichia coli* and *Staphylococcus aureus*. By measuring growth kinetics, it was found that the maximum antibacterial activity was reached after 30 h of culture, and both the optical density value at 600 nm (OD_600)_ and pH basically entered the stable phase after 20 h. Whole-genome analysis and gene cluster prediction identified a RiPP-like biosynthetic gene cluster, which comprises genes encoding precursor peptides, modification enzymes, and transport/immunity components. The molecular weight of the antimicrobial active substance was detected by dialysis and Tricine-SDS-PAGE, and it was shown to be an ultra-small molecular substance (<1 kDa). BLIS UI-11 was sensitive to protease K, but its antibacterial activity remained stable after treatment with acidic environment (pH 3.0–6.0), high-temperature treatment (121 °C for 30 min), and ultraviolet irradiation (4 h). After the sub-live cell assay (PI/SYTO9) and scanning electron microscopy (SEM), BLIS UI-11 inhibited the growth of bacteria by destroying the cell membrane of *A. hydrophila* to deform, collapse, and form holes that lead to accounting leakage. The hemolysis assay indicated that BLIS UI-11 exhibited incomplete hemolysis, suggesting its safety for application. The results showed that BLIS UI-11 produced by strain HYH-11 has great potential as an antimicrobial agent against *A. hydrophila* infection.

## Simple Summary

This study aims to explore the potential of the bacteriocin-like inhibitory substance (BLIS) UI-11 from lactic acid bacteria as a novel therapeutic agent in aquaculture. The primary objectives are to evaluate its efficacy in substituting for antibiotics against *A. hydrophila* infections and to elucidate its antibacterial mechanism. The ultimate goal of this exploration is to provide a potential candidate for reducing disease incidence and promoting healthy aquaculture practices.

## 1. Introduction

*Aeromonas hydrophila* is a Gram-negative, facultatively anaerobic, rod-shaped bacterium with a widespread distribution in aquatic environments, particularly in warm freshwater ecosystems [1,2]. As an opportunistic pathogen in fish, *A. hydrophila* is the primary etiological agent of motile aeromonad septicemia (MAS), hemorrhagic erythrodermatitis, and ulcerative syndromes, including epizootic ulcerative syndrome (EUS). Infected fish exhibit characteristic clinical manifestations, including abdominal distension and perianal bleeding [3]. Additionally, both wild and farmed fish populations are susceptible to infection [4]. Historical reports indicate that waterborne *A. hydrophila* infections represent a major etiology of high-mortality outbreaks in aquaculture systems [5]. This pathogen has induced large-scale epizootics, resulting in substantial economic losses to global aquaculture production [6]. These findings underscore the critical necessity for implementing enhanced biosecurity measures to mitigate *A. hydrophila* transmission and prevent subsequent motile aeromonosis development.

Historically, antibiotics have been the primary treatment for *A. hydrophila* infections. Although effective against bacterial pathogens, their overuse has led to serious antimicrobial resistance, posing a major threat to both ecological and public health [7]. At the same time, as bacterial resistance continues to intensify, many existing antibiotics are gradually losing efficacy. In the antibiotic supply process, insufficient quality control may allow substandard products to enter the market, further exacerbating resistance issues, increasing morbidity and mortality rates, and creating significant socioeconomic burdens [8]. Moreover, due to their unique chemical structures, antibiotics are resistant to natural degradation. They readily accumulate in organisms through the food chain and ultimately lead to exposure [9]. Their high water solubility allows antibiotics to dissolve easily in water and accumulate in fish bodies. Excessive antibiotic levels can inhibit fish growth and cause functional abnormalities in multiple organs, such as muscle, liver, kidney, and intestine [10]. As farmed fish are widely distributed through market circulation, fish carrying accumulated antibiotics may enter the human food chain, presenting a potential risk to public health. Therefore, developing safe and effective alternatives to antimicrobial drugs has become an urgent priority.

In recent years, BLIS synthesized ribosomally by lactic acid bacteria (LAB) have attracted widespread attention due to their broad-spectrum antimicrobial activity [11]. While some BLIS are primarily used as natural food preservatives in high-safety applications, others have been marketed as antimicrobial agents owing to their potent bactericidal mechanisms [12]. However, among the studies on LAB BLIS as antimicrobial agents, research specifically targeting *A. hydrophila* infections remains relatively limited. Current studies have demonstrated that LAB probiotics producing BLIS exhibit significant efficacy in treating A. hydrophila infections in fish [13]. Although numerous studies indicate that BLIS produced by LAB display notable antibacterial activity against *A. hydrophila*, the precise antimicrobial mechanisms remain to be further elucidated. Furthermore, the previous literature has repeatedly reported that BLIS produced by *L. plantarum* of Chinese origin also demonstrates remarkable antimicrobial activity [14,15]. Based on the aforementioned research background, this study successfully isolated from various fermented foods a strain of *L. plantarum* HYH-11 that produces a BLIS UI-11 with significant activity against *A. hydrophila*. Furthermore, the study was conducted on BLIS UI-11 produced by the HYH-11 strain to investigate the antibacterial activity, physicochemical properties and antibacterial mechanism of this BLIS. These findings not only provide a novel approach for the prevention and treatment of *A. hydrophila* infections but also hold significant scientific value for further exploration of the probiotic functions of LAB derived from traditional Chinese pickles.

## 2. Materials and Methods

### 2.1. Isolation and Identification of Bacteria

The strain HYH-11 was isolated from a pickled mustard tuber in Hebei Province, China. Following sampling, the tuber samples were inoculated onto De Man–Rogosa–Sharpe (MRS) agar under aseptic conditions and subsequently incubated statically at 37 °C for 24 h. Colonies exhibiting diverse morphologies were observed, from which five distinct isolates were randomly selected and purified by successive streaking. All isolates were characterized by colonial morphology and Gram staining. One isolate, designated HYH-11, was selected for further study due to its typical lactic acid bacteria morphology. It formed white, circular, smooth, convex colonies (1–2 mm in diameter) on MRS agar and was identified as Gram-positive rods. After confirming its homogeneity, the strain was mixed with 25% glycerol and stored at −80 °C. The samples were sent to a company (Shanghai Major Biomedical Technology Co., Ltd., Shanghai, China) for 16S sequencing, and the test results were compared to the NCBI database for homology comparison.

### 2.2. Evolutionary Tree of L. plantarum HYH-11

A housekeeping gene phylogenetic tree was constructed based on the sequences of 31 housekeeping genes (*dnaG*, *frr*, *infC*, *nusA*, *pgk*, *pyrG*, *rplA*, *rplB*, *rplC*, *rplD*, *rplE*, *rplF*, *rplK*, *rplL*, *rplM*, *rplN*, *rplP*, *rplS*, *rplT*, *rpmA*, *rpoB*, *rpsB*, *rpsC*, *rpsE*, *rpsI*, *rpsJ*, *rpsK*, *rpsM*, *rpsS*, *smpB*, *tsf*), using the N-J (Neighbor-joining) method in MEGA 6.0 software. Based on BLAST against a local database, 19 closely related strains were selected and a 16S rRNA phylogenetic tree was constructed using the N-J method in MEGA 6.0.

### 2.3. Preparation of L. plantarum CFS

*L. plantarum* HYH-11 was continuously shaken and cultivated in 300 mL MRS broth at 37 °C for 48 h. *L. plantarum* HYH-11 was centrifuged at 3500× *g* for 15 min using a TFL16M centrifuge (Yancheng Kate Experimental Instrument Co., Ltd., Yancheng, China). The supernatant after centrifugation was filtered through a 0.22 μm polyethersulfone microporous membrane to obtain cell-free supernatant (CFS). Concentrating the CFS tenfold using a rotary evaporator (40 °C), then filter again and store it in a refrigerator at 4 °C for subsequent experiments.

### 2.4. Basic Antimicrobial Activity Assay for CFS

To eliminate the potential interference from organic acids, the CFS was neutralized to pH 6.0 using NaOH. Catalase was added to the CFS at a final concentration of 1 mg/mL to determine the residual antibacterial activity, with the untreated CFS serving as the control. The antibacterial activity was assessed using the Oxford cup agar diffusion method. The *A. hydrophila* ATCC 7966 was grown in Brain Heart Infusion (BHI) broth at 37 °C for 12 h. Thereafter, 100 μL of the bacterial suspension (adjusted to 1 × 10^6^ CFU/mL) was spread evenly onto the surface of a Mueller–Hinton (MH) agar plate. Wells were created in the agar using an 8 mm Oxford cup, and 100 μL of CFS was added into each well. The plates were first placed at room temperature for 2 h to allow for pre-diffusion and then incubated at 37 °C for 12 h. Following incubation, the diameter of the inhibition zone was measured using a Vernier caliper.

### 2.5. Antimicrobial Spectrum

The antibacterial spectrum of the BLIS UI-11 was evaluated against a panel of bacterial pathogens using the Oxford cup method. The target strains were first cultured in their optimal media: one group (including *A. hydrophila* HYH-C12, *V. fluvialis* HYH-B7, *L. monocytogenes* ATCC 19115, *A. dhakensis* HYH-B11, *A. salmonicida* XN-9, and *A. hydrophila* ATCC 7966) in BHI broth, and the other group (comprising *S. Typhimurium* ATCC 14028, *E. coli* ATCC 25922, and *S. aureus* ATCC 25923) in LB broth. Specifically, fresh cultures were prepared by inoculating 100 µL of an overnight starter culture into the respective broth and incubating at 37 °C for 12 h, then adjusting to a 0.5 McFarland standard to obtain a suspension of approximately 1 × 10^8^ CFU/mL. These standardized suspensions were then used to prepare bacterial lawns on MH agar for the Oxford cup assay, as detailed in Section 2.4. After a further 12 h of incubation at 37 °C, the antimicrobial activity was determined by measuring the zones of inhibition against each pathogen.

### 2.6. BLIS UI-11 Production Kinetics

In order to analyze the production kinetics of BLIS UI-11 by *L. plantarum* HYH-11 production, 1 mL of overnight culture was added to 300 mL of MRS medium and incubated at 37 °C for 36 h. During the experimental period, samples were collected at regular 2 h intervals. For each time point, the following analyses were performed: the pH of the fermentation broth was measured using pH test strips, the optical density (OD_600_) of the culture was determined at 600 nm using a spectrophotometer, and the antibacterial activity of BLIS UI-11 was assessed by the agar well diffusion method.

### 2.7. Bactericidal Curve of A. hydrophila ATCC 7966 Under BLIS UI-11

In total, 40 mL of BHI medium was prepared, inoculated with 100 μL of *A. hydrophila*, and cultured until the logarithmic growth phase was reached. The culture was then divided into two 20 mL aliquots. Both aliquots were centrifuged at 3000× *g* for 5 min, after which the supernatant was discarded and the bacterial pellets were washed three times with normal saline. Subsequently, 20 mL of BLIS UI-11 and MRS medium were added to the experimental and control groups, respectively. All samples were cultured at 37 °C for 24 h. During this period, samples were taken at 2 h intervals, and bacterial growth was monitored by measuring the OD_600_. Each experiment was performed in triplicate, and the results are expressed as mean values.

### 2.8. The Stability Assay of BLIS UI-11

In order to explore the stability of the antibacterial activity of BLIS UI-11 in different environments, BLIS UI-11 was treated under different conditions of pH, temperature, proteases, and ultraviolet light, and the residual antibacterial activity was measured. For the pH stability test, seven aliquots of the sterile concentrate were adjusted to pH 3.0, 4.0, 5.0, 6.0, 7.0, 8.0, and 9.0, incubated at room temperature for 1 h, and then readjusted to pH 5.0 for activity measurement. To determine the thermal stability, six aliquots of the sterile concentrate were subjected to the following treatments: 37 °C, 50 °C, 80 °C, and 90 °C for 2 h; and 100 °C and 121 °C for 30 min. An untreated sterile concentrate was used as the control. For enzyme stability testing, five aliquots of the sterile concentrate were supplemented with 2 mg/mL of individual proteases (trypsin, proteinase K, pepsin, papain, pronase E). After incubation in a water bath at optimal temperatures for 4 h, the reactions were terminated by heating at 100 °C for 10 min. The sterile concentrate without protease was used as the control. Finally, the effect of ultraviolet light on BLIS UI-11 was assessed. Five aliquots of the sterile concentrate were taken and irradiated under an ultraviolet lamp for 0.5, 1, 2, 3, and 4 h, respectively. An unirradiated aliquot served as the control.

### 2.9. Molecular Weight Determination of BLIS UI-11 and Original Activity Assay

The BLIS UI-11 preparation was concentrated 10-fold and loaded into a 1 kDa molecular weight dialysis membrane. It was then dialyzed against deionized water for 24 h. The deionized water was changed every 6 h. Following dialysis, both the retentate (inside the membrane) and the dialysate (outside the membrane) were concentrated. Their antibacterial activity was then assessed using the Oxford cup method against *A. hydrophila* ATCC 7966 after 12 h of incubation. The molecular weight of BLIS UI-11 was determined by SDS-PAGE combined with an in situ antimicrobial activity assay. Electrophoresis was performed using a discontinuous gel system with a 4% stacking gel and an 18% separating gel. A protein molecular weight marker ranging from 2.7 to 40 kDa (Sangon Biotech, Shanghai) was used. The gel was run at 80 V for 30 min, followed by 1 h at 120 V and stained with Coomassie brilliant blue. For in situ activity analysis, the unstained gel fraction was overlaid on MH agar inoculated with 50 μL *A. hydrophila* ATCC 7966 culture and incubated at 37 °C for 12 h. The inhibitory zone was observed to confirm the antimicrobial activity of BLIS UI-11.

### 2.10. Whole Genome Analysis of L. plantarum HYH-11

Whole-genome sequencing of the bacterial strain was performed by Shanghai Major Biomedical Technology Co., Ltd. Genomic DNA was extracted using the Bacterial Genomic DNA Extraction Kit (DP302, Tiangen Biotech (Beijing) Co., Ltd., Beijing, China), and whole-genome sequencing was performed employing both the Nanopore PromethION and Illumina NovaSeq6000 platforms. Following sequencing, the quality-controlled long and short reads were hybrid-assembled with Unicycler v0.4.8, and the resulting draft genome was polished using Pilon v1.22 by mapping the Illumina short reads onto it. For genome annotation, protein-coding sequences (CDSs) were predicted with Prodigal v2.6.3, while tRNA and rRNA genes were identified using tRNAscan-SE v2.0 and Barrnap v0.9, respectively. Based on the prediction of biosynthetic gene clusters for secondary metabolites by antiSMASH v5.1.2, a genomic circle map of a single sample was generated using Circos software 0.69.6.

### 2.11. Live/Dead Cell Staining Experiment

Live/dead cell staining can effectively show the membrane damage that may be caused by BLIS UI-11. Experiments were performed according to the protocol of the SYTO-9/PI Double-stained Bacterial Viability Kit (CyberBio, Wuhan, China). Briefly, *A. hydrophila* ATCC 7966 cultured to the logarithmic growth phase was resuspended in 0.9% normal saline. Subsequently, these suspensions were incubated with BLIS UI-11 for 1 h at 37 °C. A bacterial suspension without BLIS UI-11 treatment was included as a negative control. After incubation, cells were washed three times with 0.9% normal saline and then stained with SYTO-9 and PI for 15 min in the absence of light. The stained cells were then observed under a fluorescence microscope to distinguish live (green fluorescence) from dead (red fluorescence) populations. Quantitative analysis of the fluorescence images was performed using ImageJ 1.54g software, based on which a stacked bar chart was generated using Origin 2024 SR1 software.

### 2.12. Scanning Electron Microscope (SEM)

To investigate the morphological damage inflicted by BLIS UI-11 on *A. hydrophila* ATCC 7966, bacterial cells from the exponential growth phase were harvested by centrifugation at 3500× *g* for 5 min and washed three times with PBS to remove the culture medium. The bacterial pellet was then resuspended and incubated at 37 °C for 2 h with an equal volume of BLIS UI-11 preparation. A control group was prepared by treating the bacterial cells with an equal volume of MRS broth instead of the BLIS preparation. After incubation, the cells from both treated and control groups were collected by centrifugation and fixed overnight at 4 °C with 2.5% glutaraldehyde. The fixed cells were then dehydrated through a graded ethanol series (30%, 50%, 70%, 80%, 90%, and 100%) at 4 °C, with each concentration step lasting for 15 min. The dehydrated samples were subjected to critical point drying, mounted on metallic stubs, sputter-coated with a layer of gold, and finally observed under a scanning electron microscope to examine morphological alterations.

### 2.13. The Security Testing of BLIS UI-11

To evaluate the safety of BLIS UI-11, a hemolytic activity assay was designed in this study. The experiment was performed using the blood agar plate method. A 20 μL aliquot of the test sample (BLIS UI-11) was spotted onto the surface of pre-marked blood agar plates (Bikeman Biological Technology Co., Ltd., Changde, Hunan, China) using a vertically held pipette. Phosphate-buffered saline (PBS) and *A. hydrophila* were used as the negative and positive controls, respectively. After application, the plates were incubated at 37 °C for 12 h, after which the results were observed and recorded.

### 2.14. Statistical Analysis

All data were measured in triplicate, and the final results are presented as mean ± standard deviation. One-way analysis of variance was performed using GraphPad Prism 9.5.0 software to assess statistical significance, followed by Duncan’s multiple range test for post hoc analysis. Statistical significance was defined as *p* < 0.05, with different lowercase letters indicating significant differences among parameters.

## 3. Results

### 3.1. Evolutionary Tree of HYH-11

**Figure 1:**
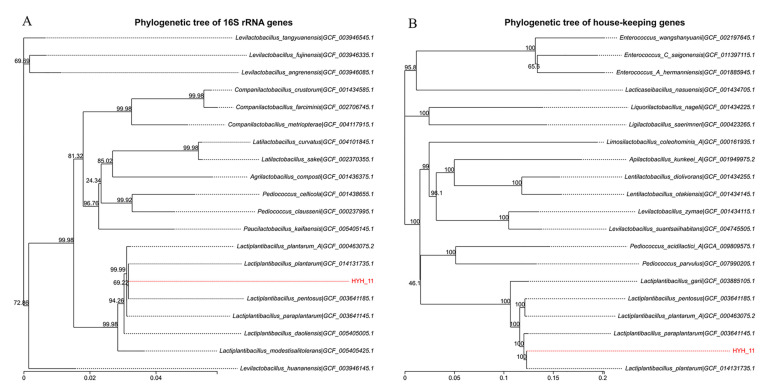
About the phylogenetic tree of sample HYH-11. (**A**) The phylogenetic tree of HYH-11 based on the 16S rRNA sequence; (**B**) Phylogenetic tree of HYH-11 based on 31 house-keeping genes. The numbers at each node of the phylogenetic tree represent the support values.

Based on BLASTn analysis of the 16S rRNA gene sequence, strain HYH-11 showed 99% sequence similarity to *L. plantarum*. To further confirm its taxonomic status, phylogenetic trees were reconstructed based on the 16S rRNA gene (Figure 1A) and housekeeping genes (Figure 1B). In both trees, strain HYH-11 formed a robust clade with reference strains of *L. plantarum*. Consequently, strain HYH-11 was identified as *L. plantarum*.

### 3.2. The Antibacterial Activity and Antibacterial Spectrum

After the addition of catalase, it was found that the CFS still exhibited antibacterial activity, and the pH value had been adjusted to exclude the influence of organic acids. Therefore, it was speculated that the CFS might produce a bacteriocin-like inhibitory substance (BLIS), and we refer to this bacteriocin-like inhibitory substance as BLIS UI-11. The antimicrobial spectrum of BLIS UI-11 was shown in Table 1, and it was found that it showed broad-spectrum antimicrobial activity against most Gram-negative and positive bacteria. Among all the indicator bacteria, BLIS UI-11 had significant antimicrobial activity against *A. hydrophila* ATCC 7966. Therefore, it is considered to have highly effective inhibitory properties against *A. hydrophila* ATCC 7966.

### 3.3. The Production Kinetics Curve of L. plantarum HYH-11 and the Inhibition Curve of A. hydrophila ATCC 7966 Under the Action of BLIS UI-11

**Figure 2:**
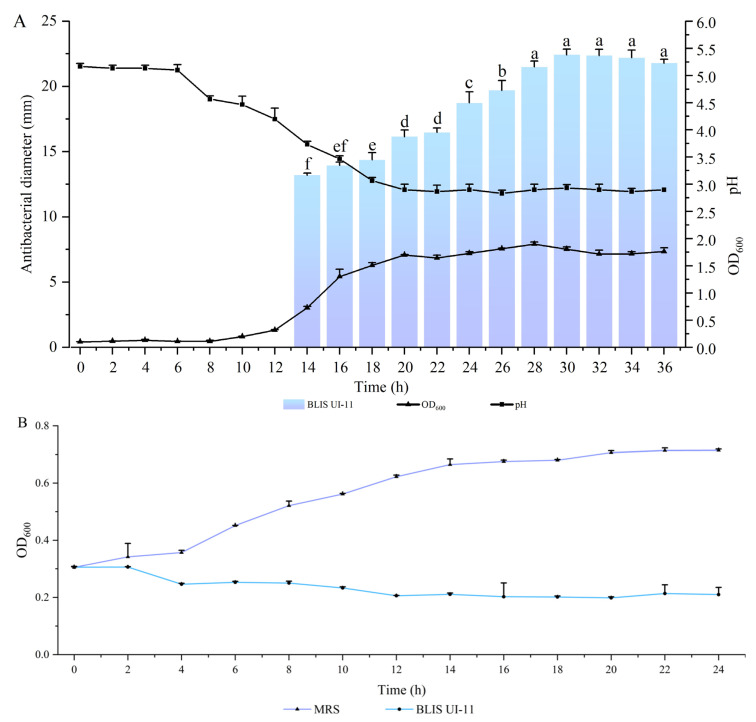
Growth curve and antibacterial activity. (**A**) Growth profile (OD_600_, black line with triangles), medium pH (black line with squares), and antibacterial activity (blue bars) of *L. plantarum* HYH-11 over time. (**B**) Inhibitory effect of BLIS UI-11 on the growth of *A. hydrophila* ATCC 7966. Bacterial growth was monitored over time after the addition of BLIS UI-11 (blue line) or an equal volume of sterile MRS broth as a control (purple line).

Growth data for *L. plantarum* HYH-11 and *A*. *hydrophila* ATCC 7966 were collected 18 times within 36 h. Based on the OD_600_ measurements in Figure 2A, the growth rate of the strain slowed down at 20 h and subsequently entered the stable phase. Regarding the acid-producing activity of *L. plantarum* HYH-11, the results showed that the pH value decreased significantly at 6 h and finally stabilized at about 3.0. Measurement of the inhibition zone data confirmed that the diameter of the inhibition zone reached a maximum at 30 h (22 mm) and the synthesis of antimicrobial substances began at 14 h (13 mm). To determine the effect of BLIS UI-11 on *A*. *hydrophila* ATCC 7966, the OD_600_ of *A*. *hydrophila* ATCC 7966 was detected within 36 h, and it was found that the addition of BLIS resulted in growth arrest of *A*. *hydrophila* ATCC 7966. These results suggest that BLIS UI-11 is able to interfere with bacterial growth, as shown in Figure 2B.

### 3.4. Effect of pH, Temperature, Protease and UV on the Antimicrobial Activity

**Figure 3:**
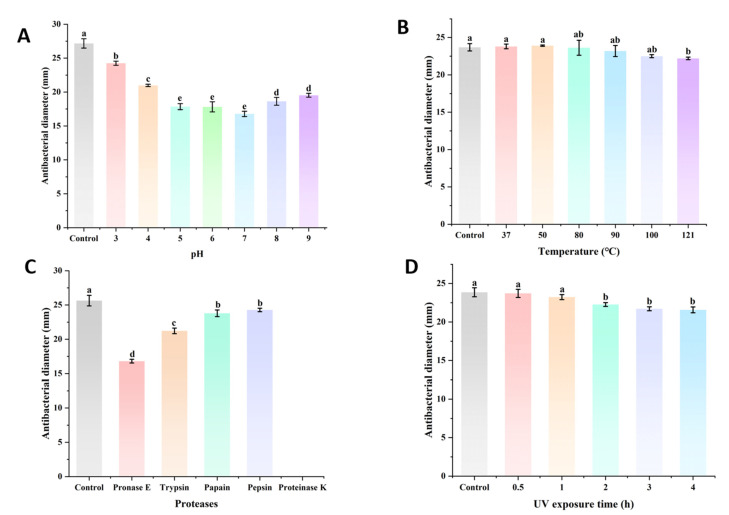
Antibacterial activity of BLIS UI-11 under different pH values, temperatures, proteases, and UV exposure. (**A**) Effect of pH on the antibacterial activity of BLIS UI-11; (**B**) Effect of temperature on the antibacterial activity of BLIS UI-11; (**C**) Effect of proteases on the antibacterial activity of BLIS UI-11; (**D**) Effect of UV exposure time on the antibacterial activity of BLIS UI-11. Each data point is the average of three replicate experiments, and the error bars represent the standard deviation.

Figure 3 illustrates the stability of *L. plantarum* BLIS UI-11 under varying pH levels, temperatures, protease treatments, and UV exposure. To evaluate the effect of pH on antimicrobial activity, BLIS UI-11 was adjusted to pH 3–9. Figure 3A showed that BLIS UI-11 showed significant antibacterial activity in a strongly acidic environment (pH 3–4), and the antibacterial activity decreased under neutral and weakly acidic conditions (pH 5–7), while the overall antibacterial activity was generally lower than that in a strongly acidic environment even though the antibacterial activity increased in the alkaline range (pH 8–9). From this, it was inferred that the BLIS UI-11 activity secreted by this strain peaked in the acidic environment. BLIS UI-11 was exposed to 37 °C, 50 °C, 80 °C, 90 °C, 100 °C, and 121 °C to explore the effect of temperature on its stability. Studies have shown that the antimicrobial activity does not change much at different temperatures, as shown in Figure 3B. In order to explore the effect of BLIS UI-11 on different proteases, proteases were mixed at a suitable temperature. Figure 3C demonstrated that the bacteriostatic zone completely disappeared following proteinase K treatment, whereas only minor inhibition was observed with other proteases. Finally, the effect of ultraviolet radiation on antibacterial activity was evaluated. Figure 3D indicated that the antibacterial activity of BLIS UI-11 remained stable throughout the 4 h treatment period.

### 3.5. The Analysis of Molecular Weight Size

**Figure 4:**
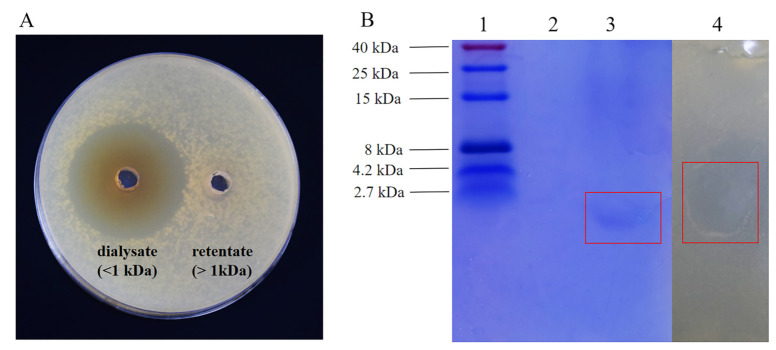
Molecular weight determination of *L. plantarum* HYH-11-generated BLIS. (**A**) Antibacterial activity of dialysate (<1 kDa) and retentate (>1 kDa); (**B**) 1: Protein band: The first lane on the polyacrylamide gel shows an ultra-low-molecular-weight protein marker; (**B**) 2: PBS blank control; (**B**) 3: Low-molecular-weight protein band showing a single protein from *L. plantarum* HYH-11; (**B**) 4: Antimicrobial test using agar (Original Figures see Supplementary Materials).

After 24 h of dialysis with the 1 kDa dialysis bag, the dialysate and retained solution were concentrated to 30 mL, respectively, and an Oxford cup was made. As shown in Figure 4A, it was found that the part injected with dialysate had an inhibition zone, but the part of the retained solution did not. Therefore, it is inferred that *L. plantarum* HYH-11 is an antimicrobial peptide less than 1 kDa. Analysis of the SDS-PAGE experiment results showed that the BLIS UI-11 extract exhibited a single protein band. Comparison with the standard protein marker revealed a molecular weight of less than 2.7 kDa. Antibacterial activity analysis of the gel bands revealed that the region corresponding to the lower molecular weight band exhibited a zone of inhibition. Both results together confirm that BLIS UI-11 is a low-molecular-weight antimicrobial peptide (Figure 4B).

### 3.6. Genomic Features and Gene Cluster Analysis of L. plantarum HYH-11

**Figure 5:**
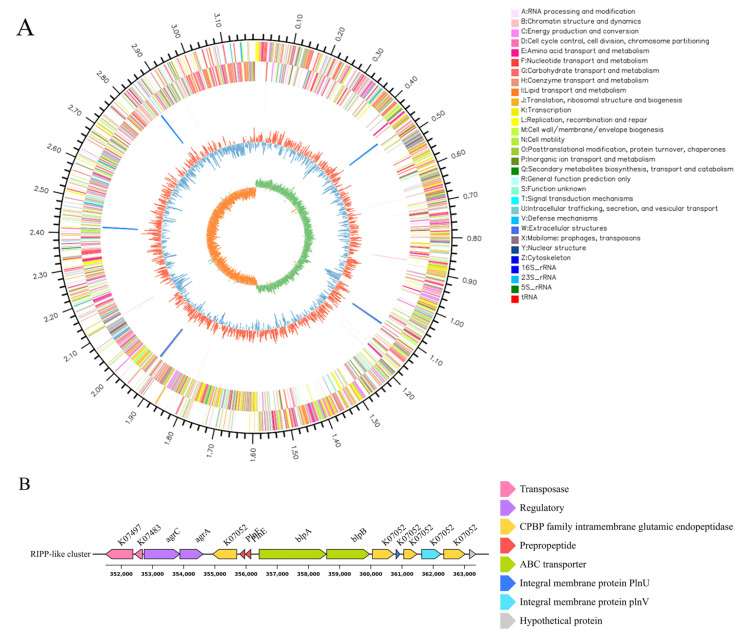
Genomic analysis of *L. plantarum* HYH-11. (**A**) Genomic Circle Map of a Single Sample. The outermost ring of the circular diagram represents the genome size. The second and third rings represent the coding sequences (CDSs) on the forward and reverse strands, respectively, with different colors indicating the functions of different CDSs. The fourth ring displays rRNA and tRNA. The fifth ring shows the GC content. The innermost ring represents the GC skew. (**B**) *L. plantarum* HYH-11 RiPP-like gene cluster. Different colors indicate different gene functions.

The genome comprises 3129 coding genes with an average GC content of 44.42%, and contains 65 tRNAs and 16 rRNAs. It includes one chromosome. The raw data have been deposited in the SRA database under BioProject ID: PRJNA1279431. A genomic circle map of a single sample was generated using Circos software. As shown in Figure 5A, the outermost ring of the circular map serves as a scale indicating genome size. The second and third rings represent CDS on the positive and negative strands, with different colors denoting the functional classification of CDS according to different COG categories. The fourth ring displays rRNA and tRNA. The fifth ring illustrates GC content: the outward red segments indicate regions where the GC content is higher than the average GC content of the entire genome, with peak heights representing the magnitude of deviation from the average; the inward blue segments indicate regions where the GC content is lower than the average, with peak heights similarly reflecting the degree of deviation. The innermost ring shows the GC-Skew value, calculated as (G − C)/(G + C). This metric helps distinguish between the leading strand (generally with GC skew > 0) and the lagging strand (generally with GC skew < 0). It also aids in identifying the origin of replication (minimum cumulative skew) and the terminus of replication (maximum cumulative skew), which is particularly crucial for circular genomes. Upon whole-genome sequencing and antiSMASH analysis of strain HYH-11, a RiPP-like gene cluster consisting of 15 genes was identified (Figure 5B). This cluster systematically integrates multiple functional units, including precursor peptide coding, post-translational modification, regulation, and transport: among these, PlnE and PlnF (two genes of unknown names that encode the antimicrobial peptide precursor proteins PlnE and PlnF, referred to here as PlnE and PlnF) are responsible for constructing the structural foundation of the antimicrobial peptides. The gene labeled K07052 encodes a key modification protein involved in the cleavage and modification of precursor peptides. The genes *agrC* and *agrA* constitute a typical two-component system that, via the quorum-sensing pathway, activate the transcription of the entire bacteriocin gene cluster in response to the accumulation of specific autoinducing peptides, and *blpA* and *blpB*, as components of an ABC transporter, are jointly responsible for the extracellular transport of BLIS and self-immunity. Through tight functional coordination, these genes collectively enable the efficient biosynthesis, regulation, and secretion of BLIS.

### 3.7. Comparison of Experimental Results and Analysis of Staining of Live and Dead Cells Under Fluorescence Microscopy

**Figure 6:**
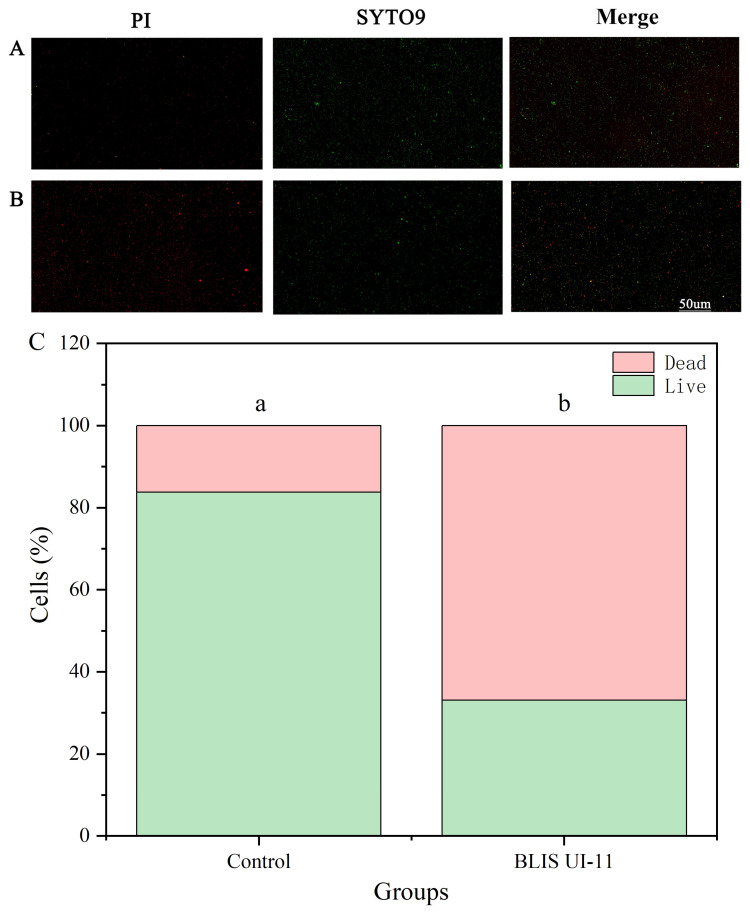
Fluorescence microscopy imaging by SYTO9 and PI staining. (**A**): Control bacteria without the addition of BLIS UI-11; (**B**) Fluorescence imaging of bacteria stained by two methods after adding BLIS UI-11 and treating for 1 h, PI (red) on the (**left**), SYTO9 (green) in the (**middle**), and combination on the (**right**). (**C**) Quantitative analysis of cell viability based on fluorescence imaging. The stacked bar chart shows the proportion of live (green) and dead (red) cells. Different letters (a, b) above the bars indicate a statistically significant difference in the proportion of dead cells between the Control (**left**) and BLIS UI-11-treated (**right**) groups.

SYTO9 is a commonly used cell membrane-permeable nucleic acid fluorescent dye that penetrates intact cell membranes, including live, dead, or fixed cells, and labels them as green fluorescent. PI, on the other hand, cannot penetrate the intact cell membrane, but is free to pass through the broken membrane and label it as red fluorescence. This experiment evaluates the bactericidal mechanism of BLIS UI-11 by the combined use of two staining modalities. Fluorescence imaging showed that both showed more green fluorescence, but the control group showed less red fluorescence (Figure 6A), and the red fluorescence intensity of the samples treated with BLIS UI-11 was significantly enhanced (Figure 6B), indicating that the cell membrane permeability of the bacteria was destroyed after BLIS UI-11 treatment. Based on this, we performed a quantitative analysis of the fluorescence imaging results and constructed a stacked bar chart (Figure 6C). The quantitative results clearly show that, compared to the control group, the percentage of dead cells in the BLIS UI-11 treatment group was significantly increased, thereby validating its damaging effect on the cell membrane from a statistical perspective.

### 3.8. Scanning Electron Microscope (SEM) Analysis

**Figure 7:**
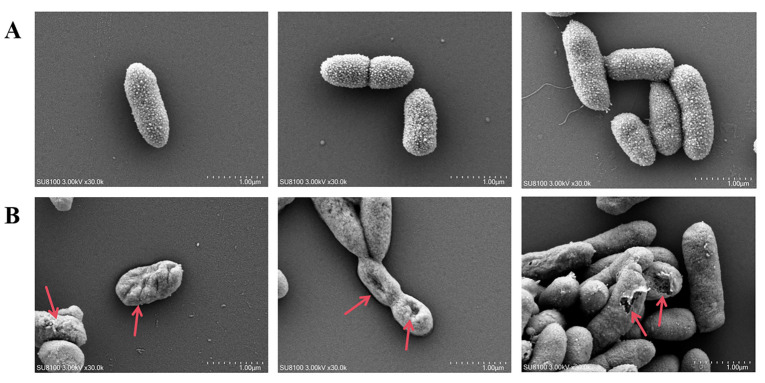
Scanning electron microscopy (SEM) results. (**A**) *A. hydrophila* ATCC 7966 treated with PBS for 1 h; (**B**) *A. hydrophila* ATCC 7966, upon 1 h of treatment with BLIS UI-11, displayed surface deformation, membrane damage, and structural collapse. The red arrow points to the place where deformation occurred.

As shown in the electron microscopy analysis of Figure 7, the cell surface of untreated *A. hydrophila* ATCC 7966 cells (control group) is intact and smooth (Figure 7A). In contrast, the cells treated with BLIS UI-11 exhibit surface deformation, membrane damage, and structural collapse (Figure 7B). From this, we deduced that BLIS UI-11 achieves bacteriostatic effects by disrupting cell membranes.

### 3.9. Hemolysis Test Measurement of BLIS UI-11

**Figure 8:**
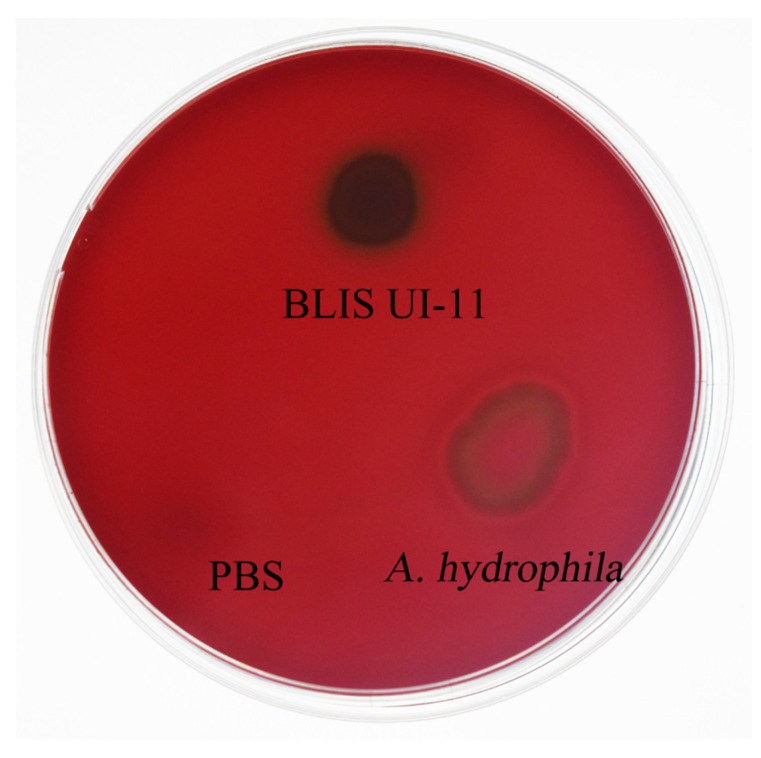
Hemolysis test results are shown. This figure represents the positive control (PBS), the negative control (*A. hydrophila* ATCC 7966), and the experimental group (BLIS UI-11).

To evaluate the in vitro hemolytic properties of BLIS UI-11, we established a standardized positive control (*A. hydrophila* ATCC 7966, inducing 100% hemolysis) and a negative control (PBS, representing the 0% hemolysis baseline). As shown in Figure 8, complete hemolysis was observed in the *A. hydrophila* ATCC 7966 positive control group, confirming the sensitivity of the experimental system. No hemolysis occurred in the PBS negative control group, which excluded non-specific interference. Under the present experimental conditions, the BLIS UI-11 treatment group resulted in the formation of a greenish hemolytic halo, but no complete hemolysis was detected. These results strongly indicate that BLIS UI-11 lacks significant hemolytic activity under the applied conditions, thereby supporting its safety profile for further evaluation and potential applications.

## 4. Discussion

The growing prevalence of *A*. *hydrophila* in recent years has posed a serious threat to aquatic animal health, causing mass mortality in aquaculture populations due to infectious diseases and thereby triggering a crisis in the aquaculture sector. In light of the fact that antibiotic overuse exacerbates antimicrobial resistance, it is imperative to develop safe and effective antibiotic alternatives for the control of *A. hydrophila*-associated aquaculture diseases.

This study demonstrates that the BLIS produced by *L. plantarum* HYH-11 exhibits potent and preferential antibacterial activity against *A*. *hydrophila* ATCC 7966. In addition, this bacteriocin exhibits significant antibacterial activity against both Gram-negative and Gram-positive bacteria, indicating that the strain displays broad-spectrum antimicrobial activity, which is consistent with previous research reports on *L. plantarum* BLIS [16]. Moreover, our investigation further revealed that the BLIS UI-11 produced by *L. plantarum* HYH-11 showed significantly stronger inhibition against *A. hydrophila* compared to foodborne strains such as *E. coli*. This specificity underscores its potential as a highly effective agent for controlling *A. hydrophila*-associated fish diseases.

Growth kinetic analysis of *L. plantarum* HYH-11 revealed that the maximum antibacterial activity was attained following 30 h of incubation. Concurrently, the pH decreased from an initial 5.3 to approximately 3.0 and stabilized after 20 h. The production of BLIS by LAB is typically growth-coupled, as shown in most studies [17].

The development of LAB BLIS as antimicrobial agents requires careful evaluation of and stability across diverse environments, a consideration well-supported by previous studies [18]. In this context, the BLIS UI-11 from *L. plantarum* HYH-11 demonstrates notable acid tolerance, retaining strong antibacterial activity under low-pH conditions. This property may be attributed to the abundance of positively charged amino acids in many LAB BLIS. Under acidic environments, the LPS in bacterial outer membranes carries increased negative charges, while the BLIS itself undergoes enhanced protonation. These effects collectively improve the BLIS’s water solubility and promote electrostatic interactions with negatively charged bacterial membranes, ultimately increasing membrane disruption efficiency [19,20]. Conversely, under alkaline conditions, alterations in the electric field reduce the protein’s surface charge and introduce charge repulsion effects. Additionally, disulfide bonds become susceptible to β-elimination, leading to disruption of the tertiary structure and destabilization of the protein [21]. Thermal stability assays revealed that BLIS UI-11 maintains excellent antibacterial activity even after 30 min treatments at 100 °C and 121 °C. This remarkable heat resistance can be attributed to several structural features: stabilizing disulfide bonds, enhanced hydrophobic interactions that maintain rigidity at high temperatures, and stabilizing post-translational modifications such as lanthionine rings or glycosylation [22,23]. In protease susceptibility tests, BLIS UI-11 was completely inactivated by proteinase K but showed resistance to other proteases. As a broad-spectrum serine protease, proteinase K cleaves both denatured and native proteins, preferentially targeting hydrophobic or aromatic amino acid residues [24]. The varying specificity of different proteases—papain with broad specificity including basic amino acid C-termini, trypsin for arginine and lysine residues, and PronaseE as a complex protease mixture containing both endo- and exopeptidases—explains the differential sensitivity pattern observed [25,26,27,28,29]. Regarding ultraviolet stability, aromatic residues in proteins can absorb UV light and induce structural damage [30]. In our experiments, a reduction in activity was observed during 2 h of UV irradiation, which may be attributed to minor protein damage in BLIS UI-11 after 2 h of exposure. Notably, the activity remained stable throughout the subsequent 3 h of irradiation. This indicates a significant degree of UV resilience in BLIS UI-11. These findings provide crucial guidance for determining optimal handling and storage conditions in potential applications, representing valuable data for their practical development.

The bacteriocin-like inhibitory substance (BLIS) UI-11 produced by *L. plantarum* HYH-11 is an ultra-low-molecular-weight bacteriocin (<1 kDa). This size is notably smaller than that of most bacteriocins from *L. plantarum*, which typically have molecular masses of 2–10 kDa, such as the ST71KS (5.0 kDa) and ZJ5 (2.5 kDa) bacteriocins [31,32]. Although a few similar ultra-small bacteriocins have been documented, such as the 618.26 Da peptide from *L. plantarum* W3-2 and the hexapeptide (761.95 Da, LNFLKK) produced by *L. plantarum* NMGL2, such low molecular weights remain uncommon among LAB [33,34].

Following the initial assessment of the antibacterial activity of the BLIS from strain HYH-11, whole-genome sequencing was performed to identify the associated BLIS gene cluster. Subsequent analysis based on these predictions focused on a RIPP-like cluster. Gene clusters responsible for BLIS biosynthesis generally comprise genes encoding a precursor peptide, modification enzymes, and transport/immunity components [35,36]. Analysis of the RIPP-like gene cluster revealed the following findings: NCBI analysis indicated that genes labeled K07497 and K07483 are associated with transposase. Two structural genes encoding the precursor peptides PlnE and PlnF yield products that are processed into two mature peptides. These precursor peptides are complementary and form the active two-peptide bacteriocin “plantaricin EF” at a 1:1 stoichiometric ratio, exhibiting synergistic antibacterial activity [37]. Studies have shown that plantaricin EF can assemble into antiparallel transmembrane helix bundles in the target cell membrane, disrupting biofilm integrity through interactions between helices, thereby exerting its antibacterial effects [38]. Further NCBI BLAST (protein) analysis demonstrated that the gene labeled K07052 encodes three proteins: the membrane-associated proteins PlnU and PlnV, and an intramembrane glutamate peptidase belonging to the CPBP family. This peptidase recognizes and binds to precursor peptides, catalyzing a cleavage reaction at the active site to remove the leader peptide and release the active core mature peptide [39], thus functioning as a modification enzyme in the gene cluster. The accessory genes *agrC* and *agrA* are involved in regulating the accessory gene regulator (agr) quorum-sensing system. As a membrane-bound receptor, AgrC recognizes specific autoinducing peptides (AIPs). Upon AIP binding, AgrC undergoes trans-autophosphorylation and subsequently transfers the phosphate group to the response regulator protein AgrA, promoting its phosphorylation or dephosphorylation. Phosphorylated AgrA binds to key promoter regions, inducing transcription of the *agr* operon and RNAIII [40]. The AgrC-AgrA complex regulates the rate of intracellular signal transduction, and its activation mechanism establishes a positive feedback loop, thereby amplifying the quorum-sensing signal [40,41]. Consequently, when bacterial density reaches a certain threshold, this system is activated and rapidly regulates transcription of the entire gene cluster. Literature reports indicate that *blpA* and *blpB* belong to the bacteriocin-like peptide (blp) system and collectively encode an ABC transporter responsible for secreting mature bacteriocins to the extracellular space to act on target bacteria [42,43]. The ATP-binding protein encoded by *blpA* forms a transport complex with the transmembrane permease encoded by *blpB*. This complex recognizes such leader sequences in an energy-dependent manner, mediates their cleavage and transmembrane transport, and ultimately releases the mature peptides into the environment [44,45]. Based on functional analysis of these genes, it is predicted that this RIPP-like gene cluster can produce class II BLIS. According to previous studies, class II BLIS are characterized as small, heat-stable antimicrobial peptides—a finding consistent with the observed heat stability and molecular weight characteristics of BLIS UI-11 [46]. Considering the presence of genes encoding PlnE and PlnF in this gene cluster, it is inferred that BLIS UI-11 is a class IIb BLIS with a small-molecular-weight peptide.

To further elucidate the mechanism of action of BLIS UI-11, growth inhibition assays, PI/SYTO9 uptake experiments, and scanning electron microscopy (SEM) were performed on *A*. *hydrophila* ATCC 7966. BLIS from LAB are known to inhibit bacterial growth primarily by disrupting cell membrane integrity or interfering with cell wall synthesis [47]. The growth inhibition results demonstrated that co-incubation of *A. hydrophila* with BLIS UI-11 significantly suppressed bacterial proliferation. Compared to the control group, the absorbance of the BLIS-treated culture remained stable over an extended period, indicating severe inhibition of bacterial growth. In the PI/SYTO9 staining assay, bacterial cells treated with BLIS UI-11 for 1 h exhibited a significantly stronger red fluorescence compared to the untreated control. Correspondingly, quantitative fluorescence analysis confirmed a significant increase in the proportion of dead cells in the treatment group. These results collectively demonstrate that BLIS UI-11 effectively disrupts the integrity of the bacterial cell membrane. The increase in PI fluorescence indicates that the cytoplasmic membrane integrity of *A. hydrophila* ATCC 7966 was compromised by BLIS UI-11. This disruption allowed PI to enter the cell and intercalate with nucleic acids, resulting in the observed fluorescence. These findings are consistent with previously reported observations [48]. Furthermore, SEM analysis confirmed that exposure to BLIS UI-11 induced significant morphological alterations in the cell surface of *A. hydrophila*, including surface deformation, membrane damage, and structural collapse. According to established classification schemes, LAB BLIS are categorized into several classes, with Class II BLIS typically acting on target cells by increasing membrane permeability, dissipating the membrane potential, and causing sublethal damage that culminates in deformation [49,50]. Based on these findings, BLIS UI-11 is proposed to inhibit bacterial growth by compromising the cytoplasmic membrane integrity of *A. hydrophila* and inducing the leakage of intracellular contents.

The safety profile of BLIS UI-11 as an antimicrobial agent was evaluated through systematic hemolysis assessment. Based on standard evaluation criteria, hemolytic responses were categorized into three types [51,52]: γ-hemolysis, characterized by the absence of any hemolytic zone around the well, indicating no hemolytic activity at the tested concentration; β-hemolysis, manifested as a clear, transparent, and colorless hemolytic zone around the well, suggesting potential hemolytic toxicity; and α-hemolysis (incomplete hemolysis), defined by an opaque greenish zone around the well, which is generally not considered indicative of typical hemolytic reactivity. The experimental results indicate that BLIS UI-11 formed a greenish hemolytic halo during testing, demonstrating characteristics of alpha-hemolysis and lacking complete hemolytic activity. The nature of alpha-hemolysis involves bacterial metabolic products such as hydrogen peroxide, which oxidize hemoglobin within red blood cells into methemoglobin, leading to the greenish discoloration of the culture medium. This process does not rupture red blood cell membranes and represents a reversible, metabolic-level interference rather than cell lysis [53]. These findings support the preliminary conclusion that BLIS UI-11 poses no significant hemolytic risk at the safety assessment level, indicating good potential for development as an aquaculture agent.

It is important to note that the conclusions of this study are derived from in vitro experiments. While these results clearly demonstrate the direct antibacterial potential of BLIS UI-11, they cannot fully reflect its true efficacy and metabolic behavior in living animals. To further evaluate its practical application potential, subsequent work should involve a challenge study in relevant fish models, focusing on the ability of BLIS UI-11 to enhance host survival and suppress the bacterial load in vivo, which is crucial for verifying its in vivo antibacterial efficacy.

However, the efficacy of BLIS UI-11 is likely to be influenced by multifactorial modulation within the complex in vivo environment of fish, including host immune responses, gastrointestinal conditions, and resident microbiota. Therefore, maintaining high survival rates and effective colonization capability of the probiotic in such intricate in vivo settings remains a pivotal challenge for translating its beneficial effects into practical applications. Future studies should focus on developing novel strategies to address these challenges. For instance, the utilization of microcapsules prepared from conjugates such as maltodextrin-sodium caseinate has been shown to effectively enhance the stability and gastrointestinal tolerance of probiotic strains [54]. Particularly noteworthy is the use of prebiotics as selective supporting substrates, which represents a highly promising enhancement strategy. As demonstrated by Barcena et al., specific prebiotics can serve as exclusive energy sources for probiotics, selectively promoting their growth and metabolic activity [55]. These prebiotics not only help maintain the viability of probiotics during in vitro culture and storage but also assist in protecting them against upper gastrointestinal tract stressors in vivo, thereby enhancing their competitiveness and persistence within complex gut microbial communities [55]. Therefore, systematically elucidating the interaction mechanisms between probiotics and prebiotics will be a critical research direction for the future.

## 5. Conclusions

In summary, this study demonstrates that *L. plantarum* HYH-11 possesses essential in vitro properties required for a probiotic. Its strong acid tolerance ensures effective survival during gastrointestinal transit. The BLIS UI-11 produced by this strain, which belongs to class IIb bacteriocins, exhibits favorable physicochemical stability and induces cell lysis and death in *A. hydrophila* by disrupting cell membrane integrity. Importantly, the biosafety of BLIS UI-11 was verified by a hemolysis assay, which demonstrated that it did not lyse mammalian erythrocytes, suggesting a high safety profile for potential therapeutic applications. Based on the aforementioned characteristics, the significant inhibitory activity demonstrated by BLIS UI-11 against *A*. *hydrophila* and other fish pathogens provides critical experimental evidence for its further development as an antibacterial agent for fish diseases. Collectively, through systematic in vitro investigations, this work lays a solid foundation for subsequent evaluation of its in vivo probiotic efficacy and potential in controlling specific bacterial diseases in fish models.

## Figures and Tables

**Table 1:** The antimicrobial spectrum of BLIS UI-11 produced by *L. plantarum* HYH-11.

Indicator Strains	Source	Gram	Inhibition Zone (mm)
**Gram-negative bacteria**			
*A. hydrophila*	ATCC 6799	−ve	31.45 ± 0.47
*A. hydrophila*	HYH-C12	−ve	30.97 ± 0.62
*A. salmonicida*	XN-9	−ve	29.67 ± 0.33
*A. dhakensis*	HYH-B11	−ve	27.13 ± 0.24
*E. coli*	ATCC 25922	−ve	24.56 ± 0.34
*S. Typhimurium*	ATCC 14028	−ve	23.41 ± 0.29
**Gram-positive bacteria**			
*S. aureus*	ATCC 25923	+ve	24.68 ± 0.56
*L. monocytogenes*	ATCC 19115	+ve	26.55 ± 0.52
*V. fluvialis*	HYH-B7	+ve	28.20 ± 0.49

Note: The diameter of the antibacterial zone covers the diameter of the Oxford cup.

## Data Availability

The original contributions presented in this study are included in the article and Supplementary Materials. Further inquiries can be directed to the corresponding authors.

## Supplementary Materials

The following supporting information can be downloaded at: https://www.mdpi.com/article/10.3390/vetsci12121165/s1, Figure S1: Original full-length gel image for Figure 4, B1, B2, B3; Figure S2: Original image of in situ activity analysis agar antibacterial test for Figure 4, B4.
