# Supplementary figures and images for: A Small-Molecular-Weight Bacteriocin-like Inhibitory Substance (BLIS) UI-11 Produced by Lactobacillus plantarum HYH-11 as an Antimicrobial Agent for Aeromonas hydrophila
Source: Vet Sci. 2025 Dec 7;12(12):1165. doi: 10.3390/vetsci12121165 (PMC12737530; doi:10.3390/vetsci12121165)

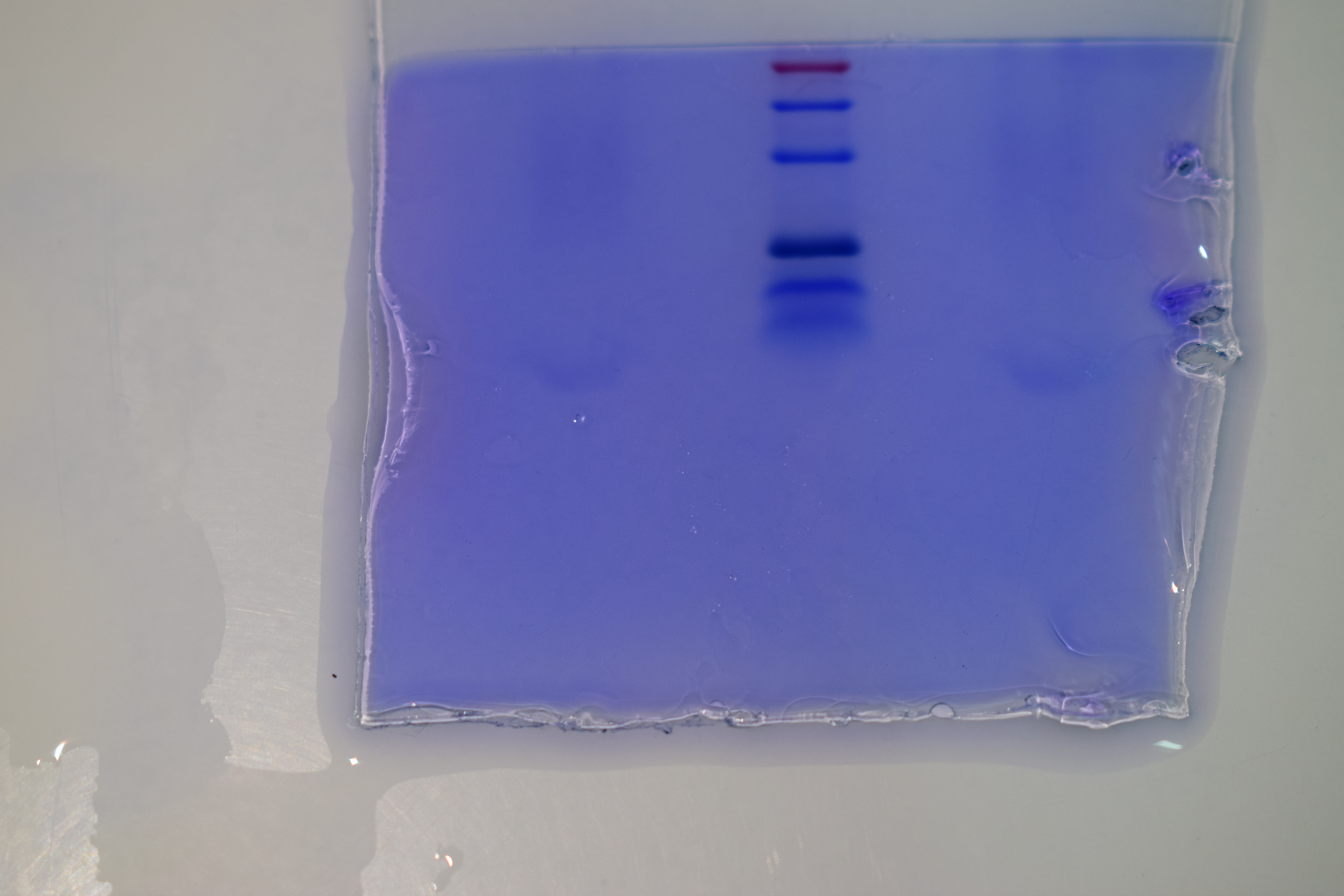

Supplement: Supplementary file 1 [file vetsci-12-01165-s001.zip › Figure S1.JPG]

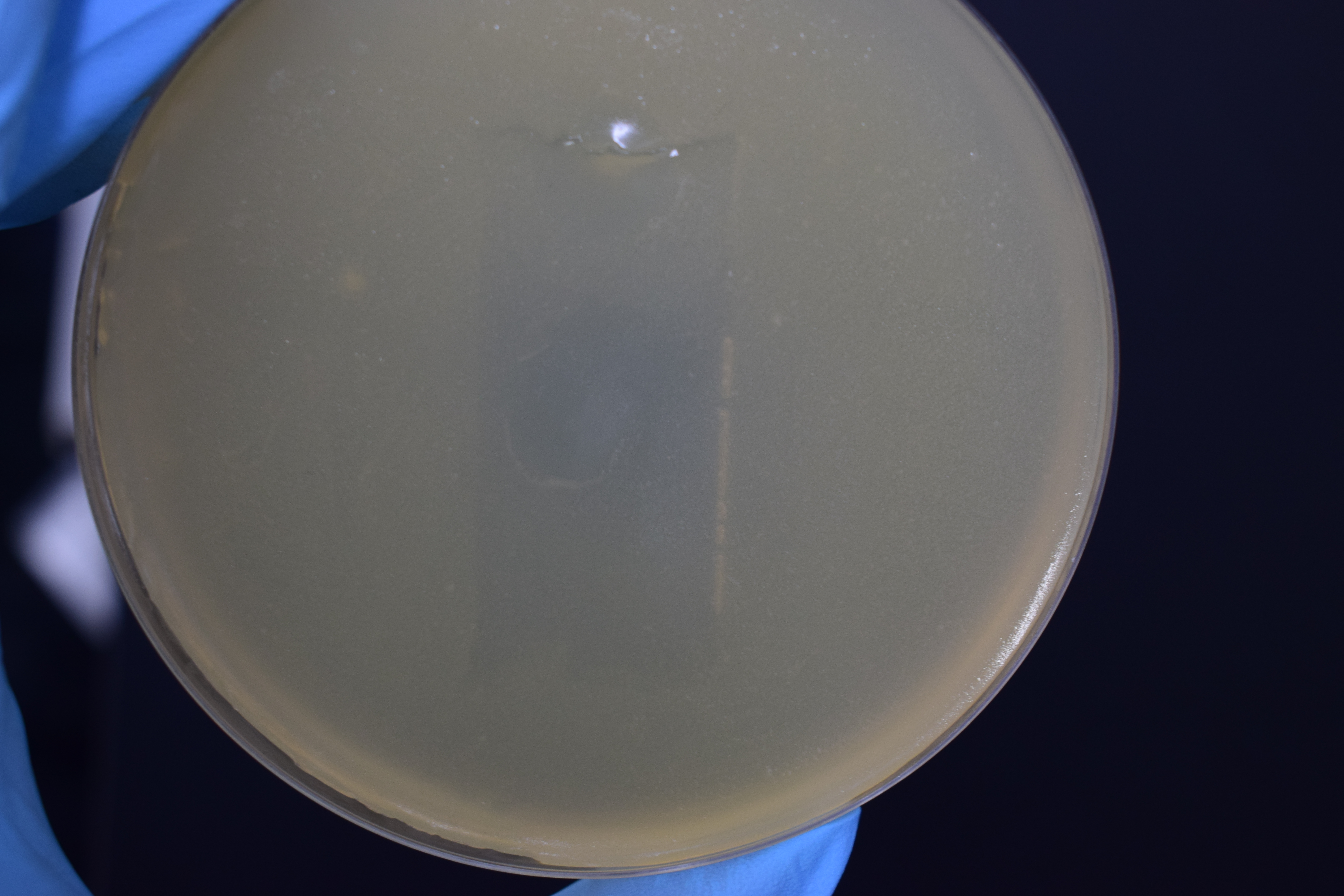

Supplement: Supplementary file 1 [file vetsci-12-01165-s001.zip › Figure S2.JPG]
